# Awareness of Tobacco-Related Health Harms among Vulnerable Populations in Bangladesh: Findings from the International Tobacco Control (ITC) Bangladesh Survey

**DOI:** 10.3390/ijerph13090848

**Published:** 2016-08-25

**Authors:** Pete Driezen, Abu S. Abdullah, Nigar Nargis, A. K. M. Ghulam Hussain, Geoffrey T. Fong, Mary E. Thompson, Anne C. K. Quah, Steve Xu

**Affiliations:** 1 Department of Psychology, University of Waterloo, Waterloo, ON N2L 3G1, Canada; geoffrey.fong@uwaterloo.ca (G.T.F.); ackquah@uwaterloo.ca (A.C.K.Q.); s4xu@uwaterloo.ca (S.X.); 2 Boston University School of Medicine, Boston Medical Center, Boston, MA 02118, USA; asm.abdullah@graduate.hku.hk; 3 Global Health Program, Duke Kunshan University, Kunshan 215347, China; 4 Duke Global Health Institute, Duke University, Durham, NC 27710, USA; 5 Economic and Health Policy Research Unit, American Cancer Society, Atlanta, GA 30303-1002, USA; nigar.nargis@cancer.org; 6 Department of Economics, University of Dhaka, Dhaka 1000, Bangladesh; think2100@gmail.com; 7 School of Public Health and Health Systems, University of Waterloo, Waterloo, ON N2L 3G1, Canada; 8 Ontario Institute for Cancer Research, Toronto, ON M5G 0A3, Canada; 9 Department of Statistics and Actuarial Science, University of Waterloo, Waterloo, ON N2L 3G1, Canada; methompson@uwaterloo.ca

**Keywords:** vulnerable populations, smoking, smokeless tobacco, health knowledge, health literacy

## Abstract

This study assessed the knowledge of the harmful effects of tobacco use among vulnerable populations in Bangladesh and whether vulnerability was associated with the presence of complete home smoking bans. Data came from Wave 3 (2011–2012) of the International Tobacco Control (ITC) Bangladesh Survey, a nationally-representative survey of 3131 tobacco users and 2147 non-users. Socio-demographic measures of disadvantage were used as proxy measures of vulnerability, including sex, residential location, education and income. Outcome measures were awareness of the harmful effects of (a) cigarette smoking and (b) smokeless tobacco use and (c) whether respondents had complete smoking bans in their homes. Logistic regression was used to examine whether the adjusted prevalence of each outcome differed by socio-demographic proxies of vulnerability. Smaller percentages of women, the illiterate, urban slum residents and low-income Bangladeshis were aware of the health harms of tobacco. These vulnerable groups generally had lower odds of awareness compared to the least disadvantaged groups. Incomplete knowledge of tobacco’s harms may prevent vulnerable groups from taking steps to protect their health. Development goals, such as increasing literacy rates and empowering women, can complement the goals of WHO’s Framework Convention on Tobacco Control.

## 1. Introduction

Social determinants of health, including income, education, occupation and residential location, are associated with cigarette smoking and tobacco use [1,2,3,4,5,6,7,8,9]. In high-income countries (HICs), smoking is more prevalent in low socioeconomic groups than high socioeconomic groups [10,11,12,13]. Such “social gradients” are also apparent in low- and middle-income countries (LMICs) [13]. Data from the Global Adult Tobacco Survey (GATS) show that the prevalence of tobacco use is highest among the least educated and the poor and decreases as education and income increase [7]. These data show that in 2009, 58% of Bangladeshis lacking a formal education used tobacco compared to only 19% of those who completed high school. Tobacco use is also higher among people living in rural areas of countries in South and Southeast Asia compared to residents of urban areas. In India, 38% of people from rural areas used tobacco in 2009 compared to 25% of people from urban areas [7]. Tobacco use is also more prevalent in urban slums: Nargis et al. [9] found that in 2009, 60% of people living in Dhaka’s urban slums used tobacco at least weekly, significantly higher than the national average of 43%.

Social determinants of tobacco use can be used to identify disadvantaged groups within society that face difficult life circumstances, such as poverty, prejudice and discrimination. These circumstances make them more vulnerable to external risks and shocks [14,15]. Residential location exacerbates these vulnerabilities. Vulnerable groups often lack support mechanisms and resources that would enhance their ability to cope with such circumstances [15,16,17].

Health literacy may enable vulnerable groups to cope with difficult life circumstances. Health literacy encompasses personal, cognitive, and social skills that allow people to understand and use information needed to improve their health [18]. Health literacy depends on essential reading and writing skills, but also on the ability to interact with health information to extract meaning, to critically analyze information and to use that information to exert control over life events. Greater literacy levels may therefore enhance personal autonomy and empowerment [18].

Key measures of health literacy used to assess the effects of health promotion interventions include knowledge and attitudes, behavioral intentions and self-efficacy [18]. From a tobacco control perspective, it is important to improve knowledge of the harmful effects of tobacco use because this knowledge is associated with intentions to quit [19,20,21,22], and intentions consistently predict future quit attempts [23]. Furthermore, it is important to understand how health knowledge may differ by socio-demographic factors in general and in vulnerable groups in particular.

Evidence from high-income countries demonstrates that knowledge of the harmful effects of cigarette smoking varies by socio-demographic factors. Studies from the United States show that sex, age, ethnicity, education, income and smoking status are all associated with awareness of the harmful effects [5,24,25,26,27]. Similar relationships were found in a survey of women from five European countries (France, Ireland, Italy, the Czech Republic and Sweden) with the additional finding that women employed in lower skilled occupations were less knowledgeable than their higher skilled counterparts [28]. Population-based surveys of smokers from Canada, the U.S., the U.K. and Australia found that having at least some university education was associated with significantly greater odds of knowing that smoking causes heart disease, stroke and impotence compared to smokers having a high school education or less. Likewise, smokers in the highest income group had significantly greater odds of knowing about these effects compared to smokers in the lowest income group [5].

Research from LMICs suggest that these same factors influence awareness of the dangers of smoking in those countries. Evidence from GATS illustrates that less educated respondents, those living in rural areas and current smokers were less aware that smoking causes stroke, heart attack and lung cancer and that second-hand smoke causes serious illness in non-smokers [29]. In Vietnam, ethnic minorities were less likely to be aware of these harms than those from the majority group [30], while in Mongolia, more educated smokers had greater odds of knowing that any amount of smoking is harmful to health compared to less educated smokers [31]. In studies of smokers from different parts of China, Yang et al. [20] and Xu et al. [32] reported significantly higher levels of knowledge of the dangers of smoking among better educated smokers (college or higher) compared to smokers having lower levels of education. In a small study of smokers from Maharashtra and Bihar, Sansone et al. [21] reported that smokers from rural areas had significantly lower knowledge of several harmful effects of smoking, including knowing that smoking causes stroke, lung cancer, premature aging and coronary heart disease. Educational attainment was also associated with knowledge: smokers in the lowest education category (illiterate or primary education only) had significantly lower odds of knowing about the harms of smoking compared to smokers having at least a college level education. Similar effects were seen among smokeless tobacco users from these same states [22].

Although some of these studies were conducted only among tobacco users [5,20,21,22,27,32], others were based on general population surveys [28,29,30,31] and therefore examined awareness of the harms of tobacco use among non-users. That non-users are aware of these harmful effects is important because their health knowledge influences their own behaviors. For example, awareness of the harmful effects of smoking can help non-smokers implement smoking bans in their homes. From this perspective, health knowledge is a form of empowerment that enables non-smokers to take action to prevent exposure to second-hand smoke. Based on a nationally-representative survey of adult Bangladeshis in 2009, Abdullah et al. [33] found that only 37% of illiterate Bangladeshis had a complete smoking ban in their home compared to 64% of Bangladeshis having nine or more years of formal education. A higher percentage of illiterate Bangladeshis also lived with two or more smokers compared to highly-educated Bangladeshis (35% vs. 26%, respectively).

The objectives of the current study were to assess knowledge of the harmful effects of tobacco use among vulnerable populations in Bangladesh in 2011–2012 using four socio-demographic measures of economic disadvantage as proxy measures of vulnerability. This study compares levels of knowledge between more and less vulnerable groups and examines the relationship between overall levels of vulnerability and health knowledge. It also examines whether vulnerability is associated with actions designed to protect personal and familial health. Since health literacy influences behavior, it is reasonable to expect that vulnerable populations having lower levels of health knowledge will be less likely to have banned smoking in their homes.

## 2. Methods

### 2.1. Sample

Data for this study came from Wave 3 of the International Tobacco Control (ITC) Bangladesh Survey, the most recent wave of the Survey to include a representative sample of urban slum residents. Data were collected from November 2011–June 2012. The ITC Bangladesh Survey is a prospective cohort survey of nationally-representative samples of tobacco users and non-users aged 15 and older. Respondents were sampled from the six administrative divisions of Bangladesh using a stratified, multi-stage sampling design. In Wave 1, 2514 tobacco users and 2120 non-users were randomly sampled from these divisions. Another 597 tobacco users and 540 non-users were sampled purposively from five of Dhaka’s urban slums. In Wave 2, respondents lost to attrition were replenished with newly-recruited respondents using the same sampling design. In Wave 3, 2703 tobacco users and 1457 non-users were followed from previous waves, while another 159 tobacco users and 148 non-users were recruited. Due to the high mobility of urban slum residents, an entirely new sample of 1055 respondents was recruited from four of Dhaka’s urban slums (because the fifth slum sampled in Wave 1 was evicted and the residents were relocated). Of these, 655 were tobacco users and 400 were non-users. In all waves, data were collected using face-to-face interviews conducted in respondents’ homes. Sampling weights were constructed so that results represent the adult population of Bangladesh [33,34,35]. The analyses presented here are based on the 5278 respondents participating in Wave 3 of the ITC Bangladesh Survey.

Data for each wave of the ITC Bangladesh Survey were collected by the Bureau of Economic Research at the University of Dhaka, in collaboration with the International Tobacco Control Project at the University of Waterloo, Canada. Sampling protocol and survey questionnaires were designed in collaboration between the ITC Project and the University of Dhaka. All survey respondents provided written informed consent prior to completing the interview, and all research protocols were approved by the institutional review board of the Bangladesh Medical Research Council (No. 1372; 19 February 2009) and the University of Waterloo Research Ethics Committee (No. 15019; 23 November 2011).

### 2.2. Measures

#### 2.2.1. Socio-Demographic Proxies of Vulnerability

As Chambers [14] notes, a myriad of life circumstances make people more susceptible to external risks and shocks. Different disciplines have defined vulnerability in different ways; economists, for example, focus on poverty, while sociologists emphasize the ability to cope with negative shocks [36]. As Adger [37] notes, vulnerability cannot be reduced to a single metric, making it difficult to quantify. Because sociologists have identified children, female-headed households, the elderly and the disabled as vulnerable groups, this study utilizes key socio-demographic factors as proxy measures of vulnerability, including sex, residence, education and income. These measures capture important aspects of vulnerability. For example, some population subgroups (e.g., women) may be vulnerable simply because of who they are, while others (e.g., the better educated) may have a greater ability to cope with negative shocks.

In this study, residence was defined as living in urban, rural or slum areas, while education was defined as having 9+ years of formal education, 1–8 years or being illiterate. Income was defined using total monthly household income, as reported by the head of each household (<5000 BDT, 5000–9999 BDT, ≥10,000 BDT, where 1 BDT = 0.0128 USD). All respondents surveyed within the same household were assigned the same value for monthly household income. For households not reporting income, income was classified as “income not reported”. Female respondents, slum residents, the illiterate and those respondents reporting monthly household incomes below 5000 BDT were considered to represent the most vulnerable groups for this analysis, while those at the opposite end of the spectrum were not vulnerable.

#### 2.2.2. Income Insecurity and Self-Rated Health

Two additional measures were used to further describe vulnerable groups. The first was a composite measure of income insecurity defined on the basis of whether respondents were able to meet five basic household needs, including needs for food, clothing, housing, healthcare and education. Possible responses included “very well”, “well”, “with difficulty” or “unable”. Response categories were reclassified into a three-point scale, where “very well” and “well” were classified as “secure” (zero), while an “unable” response was classified as “insecure” (two). Scores for the five items were then summed to yield an 11-point measure (0–10), where higher scores indicated greater income insecurity; the second measure used to describe vulnerable groups was a measure of self-rated health. Respondents were asked to describe their own health as “poor”, “average”, “good” or “excellent”. Responses were reclassified into a binary measure of self-rated health: good or excellent vs. average or poor.

#### 2.2.3. Tobacco Use

A variety of tobacco use behaviors were assessed in the ITC Bangladesh Survey. Respondents were classified on the basis of whether they smoked cigarettes, bidis (cheap, hand-rolled cigarettes common to South Asia; in Bangladesh, bidis are made from low-grade crushed tobacco rolled in white paper) or both cigarettes and bidis. Smokeless tobacco use was also assessed, as well as “mixed” use, which was defined as using both smoked and smokeless tobacco. Finally, respondents who had quit using tobacco (any and all types since being recruited to the survey) were classified as former users, while those who did not use any type of tobacco were classified as non-users. In this study, former users and non-users were collapsed into a single “non-user” category, while “dual” users (cigarette and bidi smokers) were classified as “mixed” users.

#### 2.2.4. Outcome Measures

Knowledge of the health harms of cigarette smoking and smokeless tobacco use formed the outcome measures used in this study. Respondents were asked whether cigarette smoking causes stroke, impotence in men, mouth cancer, lung cancer, coronary heart disease (CHD), chronic obstructive pulmonary disease (COPD), pulmonary tuberculosis (TB) and bronchitis. Respondents were also asked whether using smokeless tobacco causes mouth cancer, throat cancer, heart disease and gum disease. Affirmative (“yes”) responses were treated as being knowledgeable of a harm, while other responses (“no” and “don’t know”) were treated as being unaware.

Respondents were also asked whether cigarettes and smokeless tobacco contain nicotine (“Contains Nic.”) and whether nicotine is the main substance that makes people smoke (“Nic. Smoke”) or use smokeless tobacco (“Nic. Use”). Again, affirmative responses (“yes” or “true”) where treated as being knowledgeable, while other responses (“no” or “false” and “don’t know”) were treated as being unknowledgeable. Respondents were further asked whether cigarette packs, bidi packs and smokeless tobacco packaging should have more, less or the same amount of health information. These three measures were reclassified into binary variables indicating whether respondents thought each type of packaging should contain “more” health information. Since these outcomes were most relevant to current tobacco users, the analysis of these outcomes was restricted to current tobacco users, even though all respondents were asked these questions.

Finally, respondents were asked whether smoking is currently prohibited in their homes. Respondents who replied that “Smoking is not allowed in any indoor room” were classified as having complete smoking bans, while those responding “Smoking is only allowed in some rooms”, “No rules or restrictions” or “don’t know” were classified as lacking complete bans.

### 2.3. Statistical Analysis

Unless otherwise noted, all results presented here were weighted using the sampling weights; the analysis also accounted for the complex multi-stage sampling design. Descriptive statistics were used to estimate the percentage of Bangladeshis falling within each vulnerability category or to estimate the average level of income insecurity within each vulnerability indicator. Logistic regression was used to estimate the adjusted percentage of Bangladeshis (1) aware of the different health harms caused by cigarette smoking and smokeless use; (2) whether tobacco packaging should contain more health information; and (3) whether smoking is prohibited in their homes. Adjusted percentages were estimated for each socio-demographic proxy measure of vulnerability. These adjusted percentages controlled for all other covariates included in the regression models, including sampling area (non-slum areas of Dhaka vs. otherwise), tobacco use and age group. Differences in outcomes were tested between socio-demographic subgroups by comparing each subgroup against the reference group (e.g., women vs. men). These same regression models estimated the effect of each vulnerability proxy on the adjusted odds of awareness of tobacco harms, believing that tobacco packaging should contain more health information and the presence of complete smoking bans in the home. Descriptive statistics were estimated using the complex sampling routines available in SAS Version 9.4 (SAS Institute Inc., Cary, NC, USA), while logistic regression models were estimated using SAS-callable SUDAAN, Version 11.0.1 (Research Triangle Institute, Durham, NC, USA).

## 3. Results

Table 1 describes the characteristics of the sample of respondents surveyed in Wave 3. Overall, 60% of respondents were men, and 59% were under 40 years of age. Most respondents (70%) came from areas outside Dhaka, while 20% of respondents were from one of Dhaka’s urban slums sampled in Wave 3. Almost one-third of respondents were illiterate, although 43% of respondents reported monthly household incomes above 10,000 BDT. Cigarettes were the most common type of tobacco used (32%) followed by smokeless tobacco (14%). Non-tobacco users (former users and non-users) comprised 41% of the sample.

### 3.1. Characteristics of Vulnerable Populations

Table 2 examines the characteristics of vulnerable populations within Bangladesh. Similar percentages of urban and rural residents were women, while a greater percentage of slum residents were women. A similar pattern was observed for education: only 26% of Bangladeshis having nine or more years of education were women, while 43% of illiterate Bangladeshis were women. Likewise, a greater percentage of Bangladeshis not reporting their income were women compared to those having household incomes higher than 10,000 BDT per month. Illiterate Bangladeshis were older, on average, than those having at least some formal education.

Illiteracy rates were higher among slum residents compared to Bangladeshis living outside urban slums. However, living in urban slums does not necessarily correspond to lower household incomes. Only 3% of slum residents had household incomes under 5000 BDT, while a greater percentage of rural residents had incomes under 5000 BDT. In spite of this difference, income insecurity was higher among slum residents compared to urban and rural residents. Slum residents had an average income insecurity score of 3.4 points compared to scores of 1.1 and 1.7 points among urban and rural residents, respectively. Income insecurity was highest among illiterate Bangladeshis and among low-income Bangladeshis. The most vulnerable Bangladeshis tended to be in poorer health. Sixty percent of slum residents rated their health as fair or poor compared to 41% of urban residents. A similar pattern was observed by education and income, where 66% of illiterate Bangladeshis and 62% of low-income Bangladeshis rated their health as fair or poor.

Tobacco use was higher in the most vulnerable groups. Greater percentages of slum and illiterate Bangladeshis used smokeless tobacco (26% and 24%, respectively) compared to urban and highly-educated Bangladeshis (8% and 6%, respectively). Cigarette smoking was more common than other forms of tobacco use among well-educated, urban residents having monthly household incomes of 10,000 BDT or greater. A greater percentage of urban (46%) and highly-educated (52%) Bangladeshis did not use any tobacco at all compared to slum residents (36%) and illiterate Bangladeshis (32%).

### 3.2. Relationship between Socio-Demographic Vulnerability Proxies and Knowledge, Beliefs and Home Smoking Bans

Table 3 presents differences in the knowledge of the health harms of cigarette smoking by each of the four vulnerability proxies. Although there were some exceptions, the most vulnerable groups were least knowledgeable of the dangers of cigarette smoking, even after controlling for other factors. The most consistent patterns were observed for residence and education. For example, a significantly smaller percentage of slum residents knew that cigarette smoking causes CHD (53%) and COPD (46%) compared to urban residents (89% and 80%, respectively, *p* < 0.01). Similarly, 58% of slum residents knew that cigarette smoking causes impotence compared to more than 78% of urban residents (*p* < 0.05). This difference translates into an adjusted odds ratio (AOR) of 0.36 (95% CI: 0.16, 0.84). For six of the ten cigarette harm outcomes, slum residents had significantly lower odds of being aware of the harms of cigarette smoking than urban residents.

Similar differences in knowledge were observed for the education proxy. In particular, smaller percentages of illiterate Bangladeshis were aware of the health harms of cigarette smoking compared to more educated Bangladeshis. For nine of the ten measures, illiterate Bangladeshis had significantly lower odds of being aware of the harms than Bangladeshis having nine or more years of formal education (Table 3). For some outcomes, women were just as knowledgeable as men. However, a smaller percentage of women know that cigarette smoking causes impotence compared to men (62% vs. 75%, *p* < 0.001, AOR = 0.51) and that nicotine makes people smoke (20% vs. 32%, *p* < 0.001, AOR = 0.49).

Differences in knowledge by income level were less consistent. In some cases, those not reporting income were less knowledgeable (stroke, impotence, CHD, COPD, bronchitis). In other cases, the poorest were least knowledgeable (tuberculosis, nicotine makes people smoke). These results might be due to the heterogeneous nature of Bangladeshis refusing to report their income: 25% of Bangladeshis not reporting their income had nine or more years of education, while 41% were illiterate. Similarly, 31% were urban non-slum dwellers, while 36% resided in urban slums.

With respect to knowledge of the health harms of smokeless tobacco (Table 4), significantly smaller percentages of illiterate Bangladeshis knew that smokeless tobacco causes mouth cancer, throat cancer, heart disease and gum disease compared to Bangladeshis having 9+ years of education (all *p* < 0.001 and all AOR ≤ 0.37). Similarly, only 52% of urban slum residents knew that smokeless tobacco causes heart disease compared to 83% of urban non-slum residents (*p* < 0.01, AOR = 0.20). Smaller percentages of illiterate Bangladeshis knew that smokeless tobacco contains nicotine (30%) and that nicotine is what makes people use smokeless tobacco (17%) compared to better educated Bangladeshis (64% and 36%, respectively). Only 4% of slum residents knew that nicotine is what makes people use smokeless tobacco compared to 27% of urban non-slum residents (AOR = 0.10, *p* < 0.001).

Interestingly, a significantly higher percentage of slum residents knew that smokeless tobacco causes mouth cancer (97%) compared to non-slum residents (87%, *p* < 0.01). This result might be due to the higher prevalence of smokeless tobacco use among slum residents (Table 2). That is, slum residents may have had greater opportunity to be exposed to mouth cancer among family, friends and community members who were smokeless tobacco users.

When asked whether tobacco packaging should contain more health information, a different pattern of results emerged (Table 5). Although smaller percentages of women, illiterate and low-income tobacco users thought that all forms of tobacco packaging should contain more health information, a greater percentage of slum residents believed this should be the case. In all cases, tobacco users living in urban slums had 3.4–4.3-times greater odds of thinking that tobacco packaging should contain more health information compared to urban tobacco users living in non-slum areas (all *p* < 0.001). Since tobacco packaging might be the primary source of health information for these slum residents, it is possible that they may be searching for more health information. In addition, given that only 60% of illiterate tobacco users think tobacco packaging should have more health information, it is possible that text-only forms of health warnings included on tobacco packaging are not suitable for people who cannot read.

Finally, the prevalence of complete smoking bans in Bangladeshi homes was lower among vulnerable Bangladeshis. Specifically, a smaller percentage of female (52%), illiterate (51%) and low-income (50%) Bangladeshis reported having complete smoking bans in their homes (compared to 64% of men, 69% of the highly educated and 63% of high-income earners, all *p* < 0.05). Complete smoking bans were least prevalent among residents of urban slums (39%) while just under two-thirds of urban (non-slum) and rural residents reported having complete smoking bans in their homes (these differences were not statistically significant).

## 4. Discussion

This study examined associations between vulnerable populations in Bangladesh and awareness of the harmful effects of tobacco use. Rather than focusing exclusively on tobacco users, this study assessed knowledge in all groups of people because inaccurate awareness of tobacco’s harmful effects influences users and non-users alike. Using socio-demographic proxy measures of vulnerability, this study demonstrated that certain population groups are less knowledgeable of tobacco’s harmful effects, including women, the illiterate and residents of urban slums. After controlling for other factors, however, income was generally not associated with knowledge. Most vulnerable groups were significantly less likely to have complete smoking bans in their homes. Although this study relied on cross-sectional data, these findings are consistent with the notion that lower health literacy may prevent vulnerable groups from taking measures to protect their health.

Results from the logistic regression models indicate that illiteracy and residing in urban slums generally exerted similar effects on awareness of the harmful effects of tobacco use. In many cases, both of these groups have significantly lower odds of awareness than the most educated Bangladeshis or non-slum, urban residents. For certain knowledge outcomes, the magnitude of the effect is similar in both groups. In other words, illiteracy seems to exert a similar effect on knowledge as residential location. This was true for knowing that cigarette smoking causes stroke, impotence, CHD and COPD and knowing that smokeless tobacco use causes heart disease. Examination of the independent effects of socio-demographic proxies of vulnerability provides useful information for policy makers who can use the information to target media and education campaigns to specific population subgroups.

Our results are also consistent with previous studies examining awareness of the harmful effects of tobacco use among tobacco users. Findings from both HICs and LMICs indicate that educational attainment and income are associated with knowledge of tobacco’s harmful effects [5,21,22,24,25,26,27,29,31,38]. This study extends those findings, noting a more general effect of lower health knowledge among vulnerable groups irrespective of tobacco use.

These results also demonstrate that a significantly lower percentage of illiterate tobacco users thought that tobacco packaging should contain more health information compared to more educated users. In particular, illiterate tobacco smokers may be less inclined to seek out more information because they are unable to interact with the text-only information that is presented on tobacco packaging. These findings underscore the importance of pictorial health warning labels in Bangladesh as a way to increase general knowledge of tobacco’s harmful effects among tobacco users. Pictorial warning labels comprising 50% of the bottom of the pack were introduced on cigarette and bidi packaging in April 2016. Future research needs to evaluate the effects of these warnings. Moreover, public media campaigns can be used to increase knowledge among tobacco users and non-users alike.

One surprising finding is that a significantly higher percentage of tobacco users living in urban slums thought that all forms of tobacco packaging should contain more health information. Despite higher illiteracy rates, it is plausible that slum dwellers are more aware of their relative disadvantage and might be seeking ways to improve their health. As Chambers [14] cogently notes, vulnerable groups are not solely concerned with income, but with independence, mobility, security and self-respect. Wanting more information about the hazards of tobacco use may be one way that residents of Dhaka’s urban slums are trying to improve their lives.

One implication of these findings is that development goals, such as the Millennium Developmental Goals and the Sustainable Development Goals that replaced them, can enhance the goals of the World Health Organization’s Framework Convention on Tobacco Control. For example, development goals that increase literacy rates and empower women can foster the ability of these vulnerable groups to exert more control over their own personal and familial health. This includes taking steps to limit exposure to second-hand smoke by implementing home smoking bans. In this study, 51% of illiterate Bangladeshis reported having complete smoking bans in their homes compared to 69% of those having nine or more years of formal education.

One limitation of this study is that it relied on cross-sectional data to examine whether proxy measures of vulnerability were associated with knowledge of the harms of tobacco use, beliefs about health information on tobacco packaging and the presence of complete smoking bans in the home. Based on these findings, it is not possible to assess whether improving the life circumstances of vulnerable groups will provide them with better skills to protect their health. However, a small intervention study of women living in one of Mumbai’s urban slums found that knowledge of the harms of tobacco increased following participation in the educational intervention [38].

In addition, this study used socio-demographic factors as proxy measures of vulnerability. Although these measures may not capture the full spectrum of vulnerability in Bangladesh, they may identify important groups within the population that face more difficult life circumstances. For example, in patriarchal societies, such as Bangladesh, simply being female can make life circumstances more difficult, as women may have less power to make their own decisions and exert control over their health. Illiteracy not only limits employment opportunities, but makes it more difficult for people to critically interact with health information and to use it to promote health.

Two additional measures, income insecurity and self-rated health, were positively correlated with socio-demographic proxies of vulnerability. The most vulnerable groups in this study (illiterate, low-income and slum residents) had, on average, higher levels of income insecurity; smaller percentages of people in these groups rated their health as good or excellent compared to highly-educated, high-income and urban residents. Thus, although vulnerability is difficult to define, the proxy measures used in this study seem to be useful in identifying populations in Bangladesh that were more susceptible to difficult life circumstances, unhealthy lifestyles and poor health.

## 5. Conclusions

In conclusion, this study underscores the need to address disparities in tobacco control strategies among vulnerable populations in Bangladesh. These findings also have implications for other LMICs where illiteracy, poverty and urban slums are prevalent. In particular, disparities in the knowledge of tobacco-related harms translate into disparities in tobacco use prevalence and tobacco-induced morbidity and mortality. The disparities we identified between the vulnerable and non-vulnerable population in the current study should be considered as health inequities, because they are unfair and preventable [39]. Failing to address these inequities in an organized manner will widen tobacco-related knowledge gaps between vulnerable and non-vulnerable populations. In order to combat tobacco-induced disease burden in vulnerable populations, targeted preventive measures should be designed considering the socio-demographic characteristics of those populations (i.e., female gender, illiteracy and residential location).

## Figures and Tables

**Table 1:** Demographic characteristics of the respondents participating in Wave 3 of the ITC Bangladesh survey (unweighted estimates).

Characteristic	%	(Frequency)
**Sex**		
Male	59.6	(3146)
Female	40.4	(2132)
**Age Group**		
15–24	21.8	(1153)
25–39	36.9	(1947)
40–54	25.0	(1321)
55+	16.2	(857)
**Sampling Area**		
Dhaka non-slum sample	10.6	(557)
Dhaka slum sample	20.0	(1055)
Areas outside Dhaka	69.5	(3666)
**Residence**		
Urban non-slum	27.9	(1471)
Rural	52.1	(2752)
Urban slums	20.0	(1055)
**Education**		
9+ years	21.8	(1147)
1–8 years	46.0	(2423)
Illiterate	32.3	(1701)
**Income (BDT)**		
<5000	8.9	(471)
5000–9999	35.1	(1851)
≥10,000	43.1	(2274)
not reported	12.9	(682)
**Tobacco Use Status**		
Cigarettes only	31.6	(1668)
Bidis only	4.5	(236)
Dual user	3.5	(184)
Mixed user	5.3	(280)
Smokeless only	14.5	(763)
Former user	4.4	(231)
Non-user	36.3	(1916)

**Table 2:** Characteristics of vulnerable groups in Bangladesh.

	Residence			Education (Years)			Income (BDT, in 1000s)			
	Urban	Rural	Slum	9+	1–8	Illit.	≥10	5 to <10	<5	NR
**Sex**										
% female	31.9	30.9	43.9	25.8	31.1	43.0	29.7	34.1	35.8	42.0
**Age**										
Mean	37.2	37.8	36.9	33.5	34.8	43.8	38.1	37.2	39.4	35.0
**Residence**										
% slum residents	—			6.6	14.4	36.6	14.0	23.6	5.6	36.0
**Education**										
% illiterate	19.7	29.2	59.9	—			21.8	37.8	43.1	41.2
**Income**										
% low income	9.2	12.8	2.7	6.0	9.4	12.9	—			
**Income insecurity**										
Mean	1.12	1.74	3.38	0.96	1.74	2.79	0.95	2.50	3.01	2.80
**Self-rated health**										
% good/excellent	58.8	48.6	40.4	67.8	52.0	34.5	53.7	44.0	38.3	63.0
**Tobacco use**										
Cigarettes	38.3	27.7	32.2	36.1	35.0	23.7	36.1	29.7	21.3	31.1
Bidi	2.5	6.7	2.4	1.7	3.5	8.2	2.1	6.3	11.9	2.4
Mixed	5.3	13.0	3.8	4.9	8.6	12.1	9.2	11.0	8.9	3.9
Smokeless	7.6	13.4	25.5	5.7	11.6	23.6	13.8	14.9	13.6	14.0
Non-user	46.3	39.2	36.1	51.6	41.3	32.3	38.8	38.2	44.3	48.6

Illit. = illiterate; NR = not reported; Non-user: former users + non-users.

**Table 3:** Relationship between socio-demographic proxies of vulnerability and knowledge of the harms of cigarette smoking estimated using logistic regression ${}^{†}$.

	**Stroke**		**Impotence**		**Mouth Cancer**		**Lung Cancer**		**CHD**	
**Socio-Demographic**	**(*n* = 5135)**		**(*n* = 5082)**		**(*n* = 5171)**		**(*n* = 5186)**		**(*n* = 5154)**	
**Group**	**Adj %**	**(AOR)**	**Adj %**	**(AOR)**	**Adj %**	**(AOR)**	**Adj %**	**(AOR)**	**Adj %**	**(AOR)**
Sex										
Men	86.5	—	75.5	—	89.5	—	96.0	—	80.4	—
Women	85.0	(0.87)	62.4 ***	(0.51)	85.4 **	(0.67)	93.7	(0.61)	80.5	(1.00)
Residence										
Urban non-slum	90.1	—	78.4	—	92.0	—	95.5	—	89.4	—
Rural	88.8	(0.86)	72.1	(0.69)	81.9 **	(0.37)	94.4	(0.78)	87.1	(0.79)
Slum	74.9 *	(0.29)	58.5 *	(0.36)	96.0	(2.15)	96.5	(1.31)	52.7 **	(0.11)
Education										
9+ years	91.6	—	80.8	—	93.2	—	98.3	—	89.0	—
1–8 years	88.1	(0.65)	71.8 ***	(0.58)	88.6 ***	(0.55)	96.3 *	(0.44)	80.3 *	(0.44)
Illiterate	80.4 ***	(0.33)	63.4 ***	(0.38)	83.1 **	(0.34)	92.0 ***	(0.19)	75.6 ***	(0.31)
Income										
≥10,000 BDT	89.2	—	71.0	—	87.0	—	96.8	—	81.9	—
5000–9999 BDT	90.8	(1.22)	76.2	(1.34)	88.2	(1.13)	95.0	(0.60)	85.0	(1.31)
<5000 BDT	84.4	(0.63)	68.9	(0.90)	90.1	(1.39)	93.0 **	(0.42)	77.7	(0.73)
not reported	68.9	(0.23)	59.4	(0.57)	90.7	(1.49)	93.8	(0.48)	68.5	(0.40)
	**COPD**		**TB**		**Bronchitis**		**Contains Nic.**		**Nic. Smoke**	
**Socio-Demographic**	**(**n **= 5172)**		**(**n **= 5192)**		**(**n **= 5196)**		**(**n **= 5204)**		**(**n **= 5159)**	
**Group**	**Adj %**	**(AOR)**	**Adj %**	**(AOR)**	**Adj %**	**(AOR)**	**Adj %**	**(AOR)**	**Adj %**	**(AOR)**
Sex										
Men	69.2	—	95.1	—	89.4	—	51.2	—	31.7	—
Women	69.8	(1.03)	94.6	(0.90)	86.6	(0.72)	41.0	(0.62)	19.8 ***	(0.49)
Residence										
Urban non-slum	79.6	—	96.2	—	95.6	—	50.2	—	30.0	—
Rural	73.0	(0.68)	92.8	(0.50)	87.9 **	(0.29)	48.3	(0.92)	35.1	(1.29)
Slum	46.3 ***	(0.20)	98.3	(2.34)	77.5 ***	(0.12)	42.9	(0.71)	4.2 ***	(0.09)
Education										
9+ years	78.6	—	96.4	—	91.3	—	69.4	—	35.2	—
1–8 years	69.3 **	(0.58)	94.9	(0.69)	89.7	(0.81)	47.1 ***	(0.37)	28.7 **	(0.71)
Illiterate	63.5 **	(0.44)	94.0	(0.57)	85.7 **	(0.52)	33.3 ***	(0.20)	19.1 ***	(0.39)
Income										
≥10,000 BDT	68.2	—	95.3	—	91.5	—	50.0	—	31.1	—
5000–9999 BDT	75.6	(1.50)	95.5	(1.04)	94.5	(1.62)	51.3	(1.06)	25.0 *	(0.71)
<5000 BDT	69.0	(1.04)	91.4	(0.52)	88.0	(0.66)	39.7 *	(0.62)	16.9 **	(0.41)
not reported	57.9	(0.61)	95.5	(1.04)	68.1	(0.17)	38.5	(0.58)	35.2	(1.25)

Notes: * *p* < 0.05; ** *p* < 0.01; *** *p* < 0.001; test difference in the adjusted percentages between each group and the reference group (—). ${}^{†}$ Logistic regression models controlled for additional factors not shown here, including sampling area, tobacco use and age group. Adj %: model-based adjusted percentages that control for all other factors in the model. AOR: adjusted odds ratio, controlling for all other factors in the model. Contains Nic.: cigarettes contain nicotine. Nic. Use: nicotine in cigarettes makes people smoke.

**Table 4:** Relationship between socio-demographic proxies of vulnerability and knowledge of harms caused by smokeless tobacco estimated using logistic regression ${}^{†}$.

	Mouth Cancer		Throat Cancer		Heart Disease		Gum Disease		Contains Nic.		Nic. Use	
Socio-Demographic	(*n* = 5229)		(*n* = 5228)		(*n* = 5221)		(*n* = 5222)		(*n* = 5243)		(*n* = 5213)	
Group	Adj %	(AOR)	Adj %	(AOR)	Adj %	(AOR)	Adj %	(AOR)	Adj %	(AOR)	Adj %	(AOR)
Sex												
Men	90.4	—	87.5	—	78.2	—	83.5	—	46.9	—	30.2	—
Women	87.4	(0.73)	84.4	(0.76)	76.6	(0.90)	81.1	(0.84)	37.1	(0.64)	19.5 **	(0.52)
Residence												
Urban non-slum	86.7	—	86.5	—	83.1	—	85.3	—	45.0	—	27.1	—
Rural	86.8	(1.00)	83.7	(0.79)	85.4	(1.21)	82.9	(0.82)	43.3	(0.93)	34.2	(1.43)
Slum	97.4 **	(6.01)	92.5	(1.97)	52.0 **	(0.20)	79.2	(0.63)	42.3	(0.88)	4.1 ***	(0.10)
Education												
9+ years	95.5	—	93.8	—	86.4	—	89.2	—	63.9	—	35.5	—
1–8 years	90.5 **	(0.44)	87.7 ***	(0.46)	77.7 *	(0.50)	84.5 *	(0.64)	43.3 ***	(0.42)	27.5 **	(0.66)
Illiterate	82.4 ***	(0.21)	78.8 ***	(0.24)	72.5 ***	(0.36)	76.8 ***	(0.37)	29.8 ***	(0.23)	16.8 ***	(0.33)
Income												
≥10,000 BDT	89.6	—	85.6	—	79.3	—	87.0	—	45.7	—	29.2	—
5000–9999 BDT	89.6	(1.00)	86.9	(1.12)	81.9	(1.21)	88.0	(1.10)	46.5	(1.04)	24.7	(0.77)
<5000 BDT	90.9	(1.17)	87.4	(1.18)	75.4	(0.77)	78.9	(0.55)	38.9	(0.73)	16.6 **	(0.44)
not reported	87.3	(0.79)	87.3	(1.17)	64.9	(0.42)	61.5	(0.22)	33.8	(0.57)	33.2	(1.24)

Notes: * *p* < 0.05; ** *p* < 0.01; *** *p* < 0.001; test difference in adjusted percentages between each group and the reference group (—). ${}^{†}$ Logistic regression models controlled for additional factors not shown here, including sampling area, current tobacco use and age group. Adj %: model-based adjusted percentages that control for all other factors in the model. AOR: adjusted odds ratio controlling for all other factors in the model. Contains Nic.: smokeless tobacco contains nicotine. Nic. Use: nicotine in smokeless tobacco makes people use it.

**Table 5:** Relationship between socio-demographic proxies of vulnerability and opinions about (a) tobacco packaging and (b) the presence of complete bans on smoking in the home estimated using logistic regression ${}^{†}$.

Socio-Demographic Group	More Information on Tobacco Packaging ${}^{‡}$						Complete	
	Cigarettes		Smokeless		Bidis		Home Ban	
	(*n* = 3296)		(*n* = 3300)		(*n* = 3305)		(*n* = 5176)	
	Adj %	(AOR)	Adj %	(AOR)	Adj %	(AOR)	Adj %	(AOR)
Sex								
Men	72.2	—	73.5	—	71.9	—	63.8	—
Women	58.9	(0.52)	60.1	(0.51)	58.2	(0.51)	51.7 ***	(0.55)
Residence								
Urban non-slum	60.8	—	61.7	—	61.2	—	63.2	—
Rural	68.2	(1.42)	70.2	(1.52)	68.2	(1.41)	65.8	(1.14)
Slum	85.7 ***	(4.29)	85.5 ***	(4.14)	82.8 ***	(3.41)	39.3	(0.34)
Education								
9+ years	76.6	—	79.8	—	78.9	—	69.0	—
1–8 years	75.0	(0.91)	76.2	(0.80)	73.6	(0.73)	62.3 *	(0.72)
Illiterate	60.6 ***	(0.44)	61.0 ***	(0.37)	60.5 ***	(0.38)	50.7 ***	(0.42)
Income								
≥10,000 BDT	73.3	—	75.2	—	74.6	—	63.2	—
5000–9999 BDT	73.6	(1.02)	75.0	(0.99)	73.4	(0.93)	57.5	(0.76)
<5000 BDT	62.2	(0.58)	62.4 *	(0.52)	63.1 *	(0.56)	49.5 *	(0.52)
not reported	55.4	(0.43)	55.4	(0.38)	49.7	(0.31)	63.8	(1.03)

Notes: * *p* < 0.05; *** *p* < 0.001; test difference in adjusted percentages between each group and the reference group (—). ${}^{†}$ Logistic regression models controlled for additional factors not shown here, including sampling area, tobacco use and age group. Adj %: model-based adjusted percentages that control for all other factors in the model. AOR: adjusted odds ratio controlling for all other factors in the model. ${}^{‡}$ Models based on current and former tobacco users only.

## Abbreviations

The following abbreviations are used in this manuscript:

ITC	International Tobacco Control Survey
GATS	Global Adult Tobacco Survey
LMIC	Low- and middle-income county
HIC	High-income country
CHD	Coronary heart disease
COPD	Chronic obstructive pulmonary disease
TB	Tuberculosis
WHO	World Health Organization
Illit.	Illiterate
NR	Income not reported
